# Multi-clinical index classifier combined with AI algorithm model to predict the prognosis of gallbladder cancer

**DOI:** 10.3389/fonc.2023.1171837

**Published:** 2023-05-10

**Authors:** Yun Zhou, Siyu Chen, Yuchen Wu, Lanqing Li, Qinqin Lou, Yongyi Chen, Songxiao Xu

**Affiliations:** ^1^ Physical Examination Center, The Cancer Hospital of the University of Chinese Academy of Sciences (Zhejiang Cancer Hospital), The Key Laboratory of Zhejiang Province for Aptamers and Theranostics, Institute of Basic Medicine and Cancer (IBMC), Chinese Academy of Sciences, Hangzhou, Zhejiang, China; ^2^ The Clinical Laboratory Department, The Cancer Hospital of the University of Chinese Academy of Sciences (Zhejiang Cancer Hospital), The Key Laboratory of Zhejiang Province for Aptamers and Theranostics, Institute of Basic Medicine and Cancer (IBMC), Chinese Academy of Sciences, Hangzhou, Zhejiang, China; ^3^ Key Laboratory of Precision Medicine in Diagnosis and Monitoring Research of Zhejiang Province, Hangzhou, Zhejiang, China

**Keywords:** gallbladder cancer (GBC), recurrence, survival, prediction models, prognosis

## Abstract

**Objectives:**

It is significant to develop effective prognostic strategies and techniques for improving the survival rate of gallbladder carcinoma (GBC). We aim to develop the prediction model from multi-clinical indicators combined artificial intelligence (AI) algorithm for the prognosis of GBC.

**Methods:**

A total of 122 patients with GBC from January 2015 to December 2019 were collected in this study. Based on the analysis of correlation, relative risk, receiver operator characteristic curve, and importance by AI algorithm analysis between clinical factors and recurrence and survival, the two multi-index classifiers (MIC1 and MIC2) were obtained. The two classifiers combined eight AI algorithms to model the recurrence and survival. The two models with the highest area under the curve (AUC) were selected to test the performance of prognosis prediction in the testing dataset.

**Results:**

The MIC1 has ten indicators, and the MIC2 has nine indicators. The combination of the MIC1 classifier and the “avNNet” model can predict recurrence with an AUC of 0.944. The MIC2 classifier and “glmet” model combination can predict survival with an AUC of 0.882. The Kaplan-Meier analysis shows that MIC1 and MIC2 indicators can effectively predict the median survival of DFS and OS, and there is no statistically significant difference in the prediction results of the indicators (MIC1: χ^2 ^= 6.849, P = 0.653; MIC2: χ^2 ^= 9.14, P = 0.519).

**Conclusions:**

The MIC1 and MIC2 combined with avNNet and mda models have high sensitivity and specificity in predicting the prognosis of GBC.

## 1 Introduction

Gallbladder cancer (GBC) is the most invasive gastrointestinal malignant tumor in the world, with a median survival time of about six months and a five-year survival rate of less than 5% (1). It has vast geographical differences and is more common in some developing countries (2). As one of the most common biliary malignant tumors, it is a subtype with the worst prognosis and low survival time (3). The poor prognosis of GBC patients is related to tumor invasiveness, delayed diagnosis, lack of reliable biomarkers, and effective treatment. Radical surgery is the only way for patients with primary GBC to be cured (4). Surgical treatment of GBC should be performed in the medical center of experienced biliary surgeons and pathologists. The choice of operation should be based on the TNM stage of GBC (5). Patients with GBC still have a high recurrence rate after surgery (about 25%-65%) (6). Postoperative recurrence of GBC can be treated by reoperation and palliative treatment (7). Due to the originally extended resection, it is hard to extend the resection again, which leads to a poor prognosis. Therefore, the reasonable and practical assessment of recurrence and survival is the key to postoperative management.

Presently, the postoperative evaluation of GBC is mainly based on clinicopathological factors, such as TNM stage, histological type, and degree of differentiation (8). However, the clinicopathological criteria of GBC in official organizations have yet to be wholly unified (9). Pathological factors cannot fully reflect the recurrence and mortality of patients undergoing radical cholecystectomy. Finding new methods to predict recurrence and survival may help improve the prognosis management of GBC. Deepening research on dynamic monitoring of blood biomarkers can effectively evaluate the onset and progression of GBC after the operation. In analyzing targeted prediction and prognostic markers of GBC, the higher expression proportion of predictive targeting markers such as vascular endothelial growth factor and epidermal growth factor receptor in GBC come to light. Negi et al. reported that the percentage of positive lymph nodes is more capable of independently predicting the prognosis of patients undergoing radical cholecystectomy than the location or number of lymph nodes invaded (10). Masashi et al. evaluated the relationship between preoperative C-reactive protein/albumin ratio and overall survival (OS) and disease-free survival (DFS) (11). They found that the C-reactive protein/albumin ratio ≥ 0.07 was a significant independent predictor of OS, and high levels of carbohydrate antigen were significant independent predictors of DFS.

There have been studies to explore suitable biomarkers for early diagnosis, identify the molecular pathway of changes, and develop relevant biomarkers for early diagnosis, treatment, and prognosis. Despite these advances, the survival rate of patients with GBC has not improved. Since single-factor monitoring may not be able to predict recurrence and survival accurately, joint detection of multiple indicators is of great significance for the accurate assessment of the onset and progression of the disease. Our group has done previous research based on the prognosis of GBC (12). Through retrospective analysis of 260 patients with GBC who underwent radical resection, it was found that patients with high preoperative fibrinogen levels had poor DFS and OS after the operation, especially those with poor differentiation. These results suggested that fibrinogen may be a prognostic biomarker for GBC. In addition, developing various potential indicators (such as hematological markers) to form a classifier and combined with an artificial intelligence (AI) algorithm to construct a prediction model will effectively supplement the pathological factors to predict the prognosis of patients with GBC.

**Figure 1:**
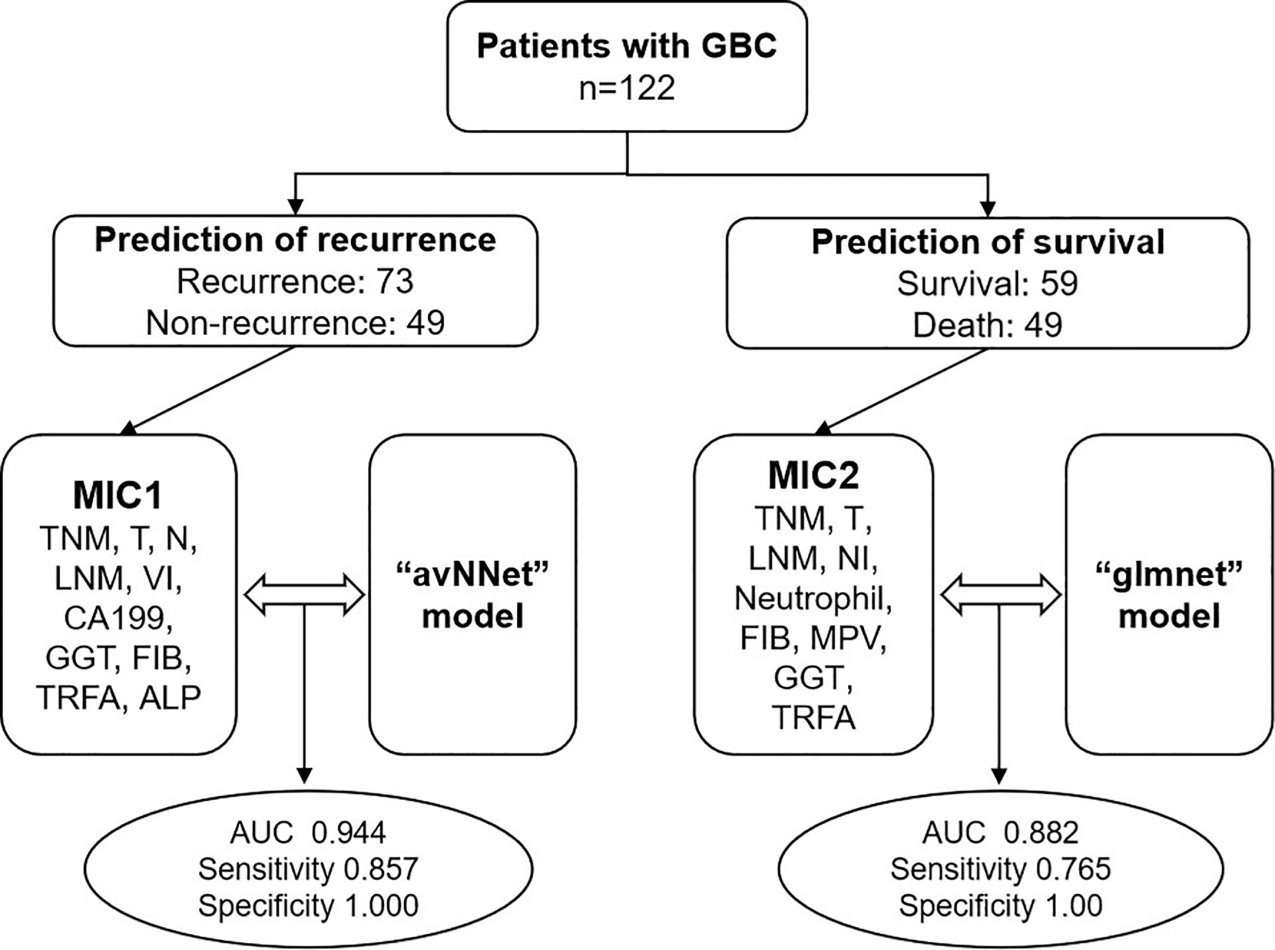
Flowchart of multi-clinical index classifier combined with AI algorithm model to predict the prognosis of GBC. TNM, TNM-stage; T, T-stage; N, N-stage; LNM, lymph node metastasis; VI, vascular invasion; NI, nerve invasion; CA199, carbohydrate antigen 199, GGT, gamma-glutamyl transpeptidase; FIB, fibrinogen; MPV, mean platelet volume; TRFA, fibrinogen/albumin; ALP, alkaline phosphatase.

To further explore the significance of various clinical indicators in the prognosis of GBC, we optimized and matched the data of 260 previous patients and finally enrolled 122 patients for this study. A comprehensive evaluation of the routine tumor markers, clinicopathological features, and blood indicators may effectively improve the prediction of the prognosis of GBC. Here, we report two models with high sensitivity and specificity for predicting GBC recurrence and survival formed by combining two multi-index classifiers (MIC1 and MIC2) with two AI algorithms (avNNet and glmnet). We aim to explore the clinical value of two models for predicting the recurrence and survival of GBC (**Figure 1**).

## 2 Materials and methods

### 2.1 Subject

A total of 122 patients with GBC from January 2015 to December 2019 were collected in this study. The admission criteria were as follows: 1) GBC diagnosed by histopathology; 2) radical cholecystectomy and postoperative pathology showed R0 margin; 3) no preoperative anticancer treatment; 4) no other malignant tumor and hematological diseases; 5) complete clinical data. This study was approved by the Ethics Committee of the Zhejiang Cancer Hospital (IRB-2022-780).

### 2.2 Data collection

In this study, the hospital information management system was used to collect original clinical data such as gender, age, height, weight, smoking history, and drinking history. Hematological parameters and pathological data were also collected. Follow-up data, including specific dates of recurrence and death, were collected through the hospital system or by telephone. Fasting blood samples were collected from all patients within one week before surgery, and the tests were performed strictly according to the instructions of the instrument and reagent.

### 2.3 Surgical strategy

All patients underwent radical resection of GBC and underwent strict imaging evaluation, which was in line with the Chinese consensus on diagnosing and treating GBC. All cases were confirmed to be R0 resection by pathology.

### 2.4 Histopathological examination

After surgical resection, the tissues were subjected to histopathological examination to collect tumor-related histopathological information. A professional pathologist performed a pathological study. According to the AJCC Cancer staging Manual, 8th edition, all cases were produced by TNM based on pathological data.

### 2.5 Follow-up data

The time between surgery and cancer recurrence was defined as DFS, and the time from surgery to death was defined as OS. The follow-up deadline was March 30, 2023. The maximum follow-up period was 80 months, and the median follow-up period was 27 months.

### 2.6 AI algorithm analysis

To enhance the usability and stability of the model, a critical evaluation is required to screen for pathological factors significantly associated with recurrence. The feature screening method uses the Boruta algorithm to estimate the contribution score of each feature in the model through the random forest strategy. The significance test can divide the features into three categories: Confirmed, Tentative, and Rejected, in which “Confirmed” is determined as an essential feature for subsequent model construction. The data is divided into a testing set and a training set. A variety of supervised classification algorithms are used to construct the prediction model. The classification algorithm includes eight algorithms: “avNNet”, “gbm”, “glmnet”, “mda”, “plr”, “svmRadial”, “naive_bayes” and “ranger”. The prediction models are constructed respectively, and the optimal model of each algorithm is obtained by using a 5-fold cross-validation method. According to the optimal model created by each algorithm, the ROC analysis method is used to evaluate the performance of the model in the testing set, and the algorithm model with the most significant AUC is selected as the final model.

### 2.7 Statistical analysis

The correlation of continuous and regular distribution variables was analyzed by independent sample t-test, and the correlation of two classification variables was analyzed by Pearson χ^2^ test. Then, the statistically significant data were assessed for relative risk and were divided into a high-risk group and a low-risk group according to the average of each index. Their p-value, risk ratio (RR), and 95% confidence interval (CI) of RR were calculated. The subjects’ working characteristic (ROC) curve was drawn by Medcalc, and the cut-off value of each index was obtained based on the Youden index. Kaplan-Meier method was used to estimate the difference between OS and DFS, and the Log Rank method and Breslow method were used to test the difference in survival analysis. SPSS was used to draw the Kaplan-Meier survival curve. The above statistics were completed by SPSS25.0 statistical software. The statistical results of bilateral P < 0.05 were considered statistically significant.

## 3 Results

### 3.1 Correlation between recurrence, survival, and clinical characteristics

A total of 122 patients and clinical characteristics in this work were detailed in **Table 1**; **Table S1**. In 122 cases with GBC, 73 (59.84%) had a postoperative recurrence, and 49 (40.16%) had no recurrence. There were 40 males (32.79%) and 82 females (67.21%). The median age was 64 years (40-92 years). The tumors in the neck and body of the gallbladder were 31 cases (25.41%) and 91 cases (74.59%), respectively. Postoperative pathological results showed that 27 patients (22.13%) were poorly differentiated and 95 (77.87%) highly differentiated. The tumor size was expressed as the maximum diameter of the tumor, with a median of 3 cm (0.6-13 cm). The vascular invasion occurred in 23 cases (18.85%), nerve invasion in 25 patients (20.49%), and lymph node metastasis in 54 cases (44.26%). The median number of regional lymph nodes was 5 (0–23). According to the pathological features, TNM staging was performed in 51 cases (41.80%) of stage I and II and 71 (58.20%) of stage III and IV.

**Table 1:** Clinical characteristics of enrolled patients with GBC.

Characteristics	N(%)	P(95%(CI))
Gender		
Male	40 (0.3278)	0.599 (−0.234 - 0.135)
Female	82 (0.6721)	
Age		
≥ 64	67 (0.5492)	0.567 (−0.124 - 0.225)
< 64	55 (0.4508)	
Scope of surgery		
Normal	111 (0.9008)	0.000 (0.345 - 0.527)
Expansion	8 (0.0655)	
Postoperative chemotherapy		
Routine chemotherapy	35 (0.2869)	0.399 (−0.406 - 0.088)
post-recurrence chemotherapy	87 (0.7131)	
Tumor location		
Neck	31 (0.2541)	0.009 (−0.423 - −0.063)
Body	91 (0.7459)	
Tumor size		
≥ 3	70 (0.5738)	0.022 (−0.375 - −0.03)
< 3	52 (0.4262)	
Differentiation		
Low	27 (0.2213)	0.388 (−0.296 - 0.117)
High	95 (0.7787)	
PVI, HAI		
Yes	6 (0.0492)	0.000 (−0.528 - −0.348)
No	116 (0.9508)	
T		
T3+T4	45 (0.3689)	0.000 (−0.61 - −0.324)
T1+T2	77 (0.6311)	
N		
N1+N2	49 (0.4016)	0.000 (−0.562 - −0.252)
N0	73 (0.5984)	
M		
M1	7 (0.0574)	0.096 (−0.639 - 0.065)
M0	115 (0.9426)	
TNM		
III+IV	71 (0.5820)	0.000 (−0.699 - −0.402)
I+II	51 (0.4180)	
LNM		
Metastasis	54 (0.4426)	0.000 (−0.562 - −0.252)
Normal	68 (0.5574)	
RLN count		
≥ 5	73 (0.5984)	0.108 (−0.322 - 0.032)
< 5	49 (0.4016)	
Vascular invasion		
Yes	23 (0.1885)	0.002 (−0.49 - −0.119)
No	99 (0.8115)	
Nerve invasion		
Yes	25 (0.2049)	0.002 (−0.473 - −0.107)
No	97 (0.7951)	

PVI, portal vein invasion; HAI, hepatic artery invasion; T, T-stage; N, N-stage; M, M-stage; TNM, TNM-stage; LNM, lymph node metastasis; RLN, regional lymph node.

**Table S2** shows 26 clinical indicators related to patients’ recurrence. The results also show that recurrence is unrelated to sex, age, and degree of differentiation. However, it relates to the TNM stage, operation scope, tumor site, lymph node metastasis, and nerve and vascular invasion. In short, patients with late TNM stage (P < 0.001), lymph node metastasis (P < 0.001), and high levels of alkaline phosphatase (ALP) (P < 0.001) were significantly correlated with recurrence. **Table S3** shows 22 clinical indicators related to patients’ survival. The results show that patients with late TNM stage (P < 0.001), lymph node metastasis (P < 0.001), and high levels of fibrinogen (FIB) (P = 0.001) and ferritin (FER) (P = 0.001) were significantly correlated with recurrence.

### 3.2 The filtering of candidate indicators of MIC1 and MIC2 in clinical characteristics

The relative risk analysis of recurrence and death was carried out for the factors related to the recurrence outcome. The results of TNM staging, T staging, N staging, lymph node metastasis (LNM), tumor in the gallbladder neck, non-extended surgical range, vascular and nerve invasion, high level of FIB, fibrinogen/albumin (TRFA), carbohydrate antigen 125 (CA125), carbohydrate antigen 199 (CA199), glutamic oxalacetic transaminase (AST), FER, direct bilirubin (DBIL), total bile acid (TBA), ALP, gamma-glutamyl transpeptidase (GGT), total bilirubin (TBIL), neutrophil, and thrombocytocrit (PCT), a total of 22 clinical factors are risk factors for recurrence of GBC (**Table S4**). Based on the ROC analysis of the 23 risk factors for recurrence prediction, 14 risk factors with AUC > 0.5 and P < 0.05 were selected as the MIC1 candidate indicators for predicting recurrence (**Table 2**).

**Table 2:** ROC analysis of MIC1 candidate indicators for recurrence in patients with GBC.

Factor	AUC	P	95%CI	
			Low	High
TNM	0.780	0.000	0.696	0.865
CA199	0.759	0.000	0.674	0.844
GGT	0.734	0.000	0.645	0.823
LNM	0.722	0.000	0.634	0.809
T	0.719	0.000	0.631	0.808
FIB	0.710	0.000	0.616	0.804
TRFA	0.706	0.000	0.612	0.800
N	0.706	0.000	0.614	0.797
FER	0.685	0.000	0.592	0.777
ALP	0.658	0.001	0.565	0.751
CA125	0.650	0.002	0.556	0.745
PCT	0.636	0.006	0.539	0.733
Neutrophil	0.629	0.009	0.532	0.727
NI	0.599	0.046	0.502	0.697
Site	0.598	0.051	0.500	0.696
VI	0.596	0.055	0.498	0.693
Size	0.594	0.067	0.493	0.694
AST	0.586	0.087	0.488	0.684
Scope of surgery	0.564	0.211	0.464	0.663
DBIL	0.559	0.244	0.460	0.658
TBA	0.555	0.284	0.455	0.655
TBIL	0.553	0.295	0.454	0.652
PVI, HAI	0.547	0.360	0.447	0.647

TNM, TNM-stage; CA199, carbohydrate antigen 199; GGT, gamma-glutamyl transpeptidase; LNM, lymph node metastasis; T, T-stage; FIB, fibrinogen; TRFA, fibrinogen/albumin; N, N-stage; FER, ferritin; ALP, alkaline phosphatase; CA125, carbohydrate antigen 125; PCT, thrombocytocrit; NI, nerve invasion; VI, vascular invasion; AST, glutamic oxalacetic transaminase; DBIL, direct bilirubin; TBA, total bile acid; TBIL, total bilirubin; PVI, portal vein invasion; HAI, hepatic artery invasion.

The same method was used to screen risk factors for predicting death from data from 108 patients. The results showed that advanced TNM staging, late T staging, late N staging, lymph node metastasis, high regional lymph node count, nerve invasion, high level of FIB, TRFA, CA125, FER, ALP, GGT, neutrophil count, white blood cell count (WBC) and PHOS, a total of 16 clinical detection factors are risk factors for predicting survival in patients with GBC within five years (**Table S5**). Based on the ROC analysis of the 16 risk factors, 15 risk factors with AUC > 0.5 and P < 0.05 were selected as the MIC2 candidate indicators to predict survival (**Table 3**).

**Table 3:** ROC analysis of MIC2 candidate indicators for survival in patients with GBC.

Factor	AUC	P	95%CI	
			Low	High
TNM	0.812	0.000	0.705	0.919
T	0.761	0.000	0.646	0.877
FIB	0.747	0.000	0.625	0.869
TRFA	0.737	0.000	0.614	0.860
GGT	0.705	0.001	0.583	0.827
FER	0.705	0.001	0.583	0.827
PHOS	0.693	0.003	0.567	0.819
Neutrophil	0.689	0.003	0.565	0.814
CA125	0.683	0.005	0.556	0.809
LNM	0.679	0.005	0.553	0.806
N	0.659	0.016	0.530	0.789
WBC	0.652	0.022	0.522	0.782
RLN	0.646	0.029	0.515	0.777
NI	0.636	0.042	0.505	0.767
MPV	0.633	0.054	0.498	0.767

TNM, TNM-stage; T, T-stage; FIB, fibrinogen; TRFA, fibrinogen/albumin; GGT, gamma-glutamyl transpeptidase; FER, ferritin; CA125, carbohydrate antigen 125; LNM, lymph node metastasis; N, N-stage; WBC, white blood cell count; RLN, regional lymph node; NI, nerve invasion; MPV, mean platelet volume; ALP, alkaline phosphatase.

### 3.3 Evaluation of the recurrence prediction power of MIC1 combined AI algorithm in GBC

**Figure 2:**
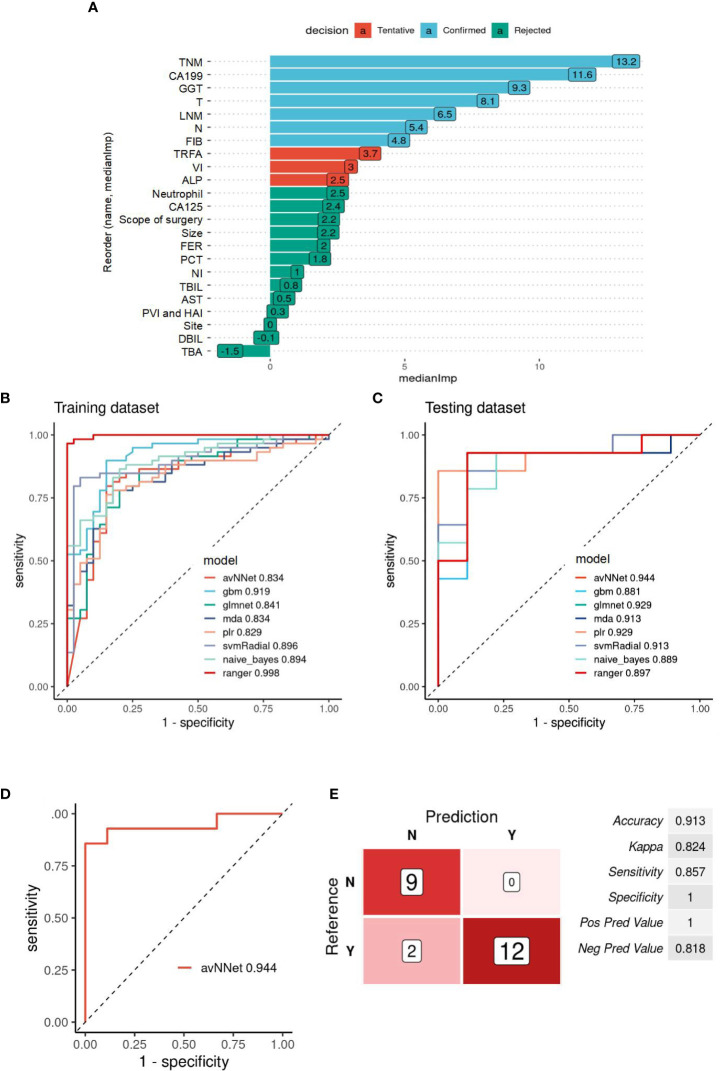
Evaluation of the recurrence prediction power of MIC1 combined AI Algorithm in GBC. **(A)** Statistical plot of the contribution of each feature in the model estimated by the random forest strategy. By testing the significance obtained, the features can be classified into three categories: Confirmed, Tentative, and Rejected. “Confirmed” is selected as an important feature for MIC1 modeling analysis. **(B)** ROC curves of the MIC1-based optimal models constructed by the nine algorithms in the training and **(C)** testing datasets. **(D)** ROC curves of the MIC1-based “avNNet” model in testing datasets, AUC=0.944. **(E)** The evaluation indicators of MIC1-based “avNNet” model prediction efficacy. TNM, TNM-stage; CA199, carbohydrate antigen 199; GGT, gamma-glutamyl transpeptidase; T, T-stage; LNM, lymph node metastasis; N, N-stage; FIB, fibrinogen; TRFA, fibrinogen/albumin; VI, vascular invasion; ALP, alkaline phosphatase; CA125, carbohydrate antigen 125; FER, ferritin; PCT, thrombocytocrit; NI, nerve invasion; TBIL, total bilirubin; AS, glutamic oxalacetic transaminase; DBIL, direct bilirubin; TBA, total bile acid.

Fourteen MIC1 candidate indicators of 122 patients were used to construct a predictive model to evaluate the risk of recurrence. “Confirmed” are selected by the Boruta algorithm, and these ten “Confirmed” features are set as MIC1 for modeling and analysis (**Figure 2A**). The patients were divided into a training dataset and a testing dataset for the proportion of 8:2. According to the optimal model constructed by each algorithm, the ROC analysis method is used to evaluate the performance of the model in the testing set, and the algorithm model with most significant AUC is selected as the final model. The ROC curves of the optimal model constructed by the nine algorithms in the training and testing dataset are shown in **Figures 2B, C**. The results show that the MIC1-based model constructed by the avNNet algorithm has the highest AUC of 0.944 in the testing set, and the model is selected as the final recurrence risk prediction model (**Figure 2D**). The ROC curve was drawn with the predicted value in the testing set, and the best diagnostic cut-off value was set to 0.255 according to the Youden-index. When the predictive value of the diagnostic model is less than 0.255, it is considered low risk (no recurrence) within four years. When the model’s predictive value is more than 0.255, it is regarded as high risk (recurrence), and the evaluation indicators to obtain the predictive efficiency of the model are shown in **Figure 2E**. The results show that the accuracy, sensitivity, and specificity of predicting recurrence in GBC by the MIC1-based model are 0.913, 0.824, and 0.857, respectively.

### 3.4 Evaluation of the survival prediction power of MIC2 combined AI algorithm in GBC

**Figure 3:**
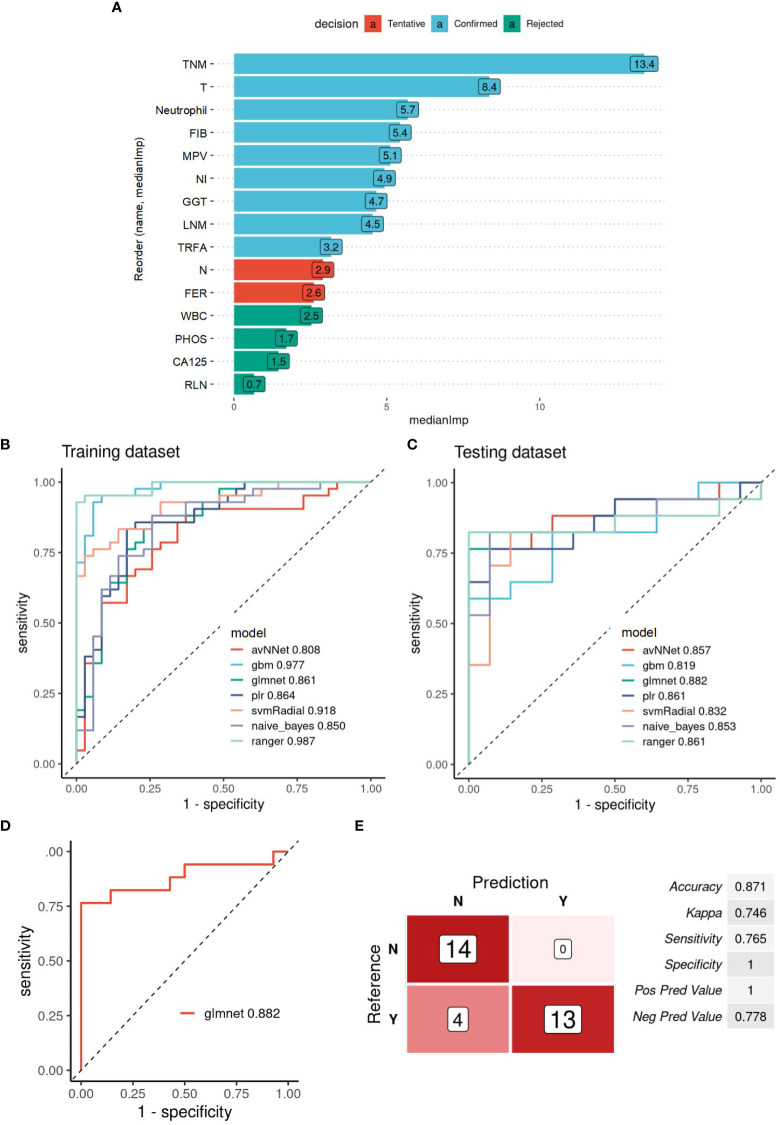
Evaluation of the survival prediction power of MIC2 combined AI Algorithm in GBC. **(A)** Statistical plot of the contribution of each feature in the model estimated by the random forest strategy. By testing the significance obtained, the features can be classified into three categories: Confirmed, Tentative, and Rejected. “Confirmed” is selected as an important feature for MIC2 modeling analysis. **(B)** ROC curves of the MIC2-based optimal models constructed by the nine algorithms in the training and (C.) testing datasets. **(D)** ROC curves of the MIC2-based “glmnet” model in testing datasets, AUC=0.882. **(E)** The evaluation indicators of MIC2-based “glmnet” model prediction efficacy. TNM, TNM-stage; T, T-stage; FIB, fibrinogen; MPV, mean platelet volume; NI, nerve invasion; GGT, gamma-glutamyl transpeptidase; LNM, lymph node metastasis; TRFA, fibrinogen/albumin; N, N-stage; FER, ferritin; WBC, white blood cell count; CA125, carbohydrate antigen 125; RLN, regional lymph node.

The same method was used to construct a prediction model based on the information of 15 MIC2 candidate indicators to evaluate the survival risk of patients. Nine “Confirmed” features are selected as MIC2 for modeling and analysis (**Figure 3A**). According to the proportion of 7:3, the patients were divided into training and testing datasets. The ROC curves of the optimal model constructed by the nine algorithms in the training set and testing set are shown in **Figures 3B, C**. The MIC2-based model constructed by the “glmnet” algorithm has the highest AUC of 0.882 in the testing dataset, and the model is selected as the final survival risk prediction model (**Figure 3D**). Set the optimal diagnostic cut-off value to 0.331 according to the Youden-index value. When the predicted value of the diagnostic model is less than 0.331, the patient to be tested is considered low risk (survival) within four years. When the model’s predicted value is more than 0.331, the patient to be tested is regarded as high risk. The model’s prediction efficiency evaluation indicators are obtained, as shown in **Figure 3E**. The results show that the accuracy, sensitivity, and specificity of predicting recurrence in GBC by the MIC2-based model are 0.871, 0.765, and 1, respectively.

### 3.5 Kaplan-Meier analysis of MIC1 and MIC2 estimation power of DFS and OS in GBC

The MIC1 and MIC2 estimation power of DFS and OS were analyzed by Kaplan-Meier. Kaplan-Meier analysis of MIC1 indicators predicted DFS in 122 patients (**Table S6**). The DFS of patients with late TNM stage, late T stage, late N stage, vascular invasion, lymph node metastasis, and high levels of CA199, FIB, and TRFA were worse than patients with a low level of them (**Figure S1**). However, there was no significant difference in DFS predicted by high levels of GGT and ALP. Kaplan-Meier analysis of MIC2 indicators predicted OS in 108 patients similarly (**Table S7**). The results show that the OS of patients with advanced TNM stage, late T stage, lymph node metastasis, nerve invasion, high FIB, MPV, and TRFA are worse than patients with a low level of them (**Figure S2**). The results suggest that when evaluating the OS and DFS of GBC patients, personalized management can be done by comprehensively analyzing the MIC1 and MIC2 indicators.

## 4 Discussion

Although many new chemotherapeutic drugs are produced and used to treat GBC, surgical resection is still the most effective. However, the recurrence and low survival rate after resection of GBC is still a complex problem that modern medicine cannot overcome. Usually, early diagnosis of GBC and timely and effective treatment can improve the five-year survival rate to 75% (1). Currently, the clinic can only manage the postoperative prognosis according to the basic pathological information, such as a clinical stage. However, the repeated adjustment of AJCC staging criteria still does not effectively affect the prognosis judgment (13). The lack of pragmatic markers to identify patients with poor prognoses may be challenging to manage forecasts effectively. Many meaningful research results have been reported to solve the problem of predicting the prognosis of GBC.

Although conventional tumor markers such as CA199, CA125, and CEA are prognostic indicators of GBC, they are still not ideal as a single index to predict the prognosis of GBC due to the lack of specificity of GBC (14). Cui et al. analyzed the clinical information such as neutrophil-lymphocyte ratio (NLR), monocyte-lymphocyte ratio (MLR), and ALP. It was only confirmed that NLR before treatment was an independent prognostic factor and biomarker of poor OS in GBC patients with different treatments (15). But what is interesting is that the predictive value of MLR in patients with GBC after the operation is related to age. There is a significant difference in the cut-off value of MLR between ≤ 60 years old and > 60 years old in patients with GBC (16). In our study, ALP (RR=1.495, 95% CI: 1.145-1.952) and GGT (RR=1.472, 95%CI:1.126-1.925) were risk factors for the recurrence of GBC. Pancreatic biliary reflux is a risk factor for GBC and cholangiocarcinoma, so the early diagnosis and treatment of pancreatic juice reflux plays a vital role in preventing GBC and cholangiocarcinoma (17). Studies have shown that GGT and ALP significantly increase in pancreatic and biliary reflux patients (18). Our results also showed that ferritin was a risk predictor of recurrence (RR=1.698, 95% CI: 1.302-2.215 (*P* < 0.001)). Because cancer cells can produce synthesis and secrete ferritin, cancer cells affect the uptake and clearance of ferritin. When cells are damaged and necrotic, the ferritin stored in the cytoplasm will flow into the blood, increasing ferritin. Nerve infiltration and lymphatic vascular invasion of GBC are independent risk factors for early recurrence of T-staged advanced GBC after radical resection (19, 20). Wang et al. reported that lymph node metastasis and platelet count were predictors of OS (21). Yang et al. found that tumors in the neck of the gallbladder significantly increased the difficulty of the operation and reduced the chance of radical resection. Gallbladder neck tumors can independently predict poor prognosis (22). In addition, the increase of preoperative fibrinogen-specific albumin was significantly correlated with the negative OS rate of GBC patients. The growth of preoperative albumin level was a prognostic factor for GBC patients. The best critical value of the preoperative fibrinogen-specific albumin ROC curve was 0.08 (23). FIB plays a key role in the coagulation pathway and in the coagulation cascade (24). Elevated plasma fibrinogen levels reflect the hypercoagulable state and thrombophilia induced by tumor cells (25). In addition, FIB has been shown to be associated with adverse clinical outcomes in various types of cancers such as gastric, cervical, colorectal, ovarian, and urothelial cancers (26–29). Serum ALB levels can reflect systemic inflammatory response and nutritional status, and reduced ALB levels have been shown to be associated with poor prognosis in a variety of cancers (30). The predictive effect of TRFA is more sensitive in predicting the prognosis of patients with malignant tumors (31). Many of these research results are also reflected synchronously in the risk factor analysis part of this study.

Although there are many studies on prognostic risk factors, few studies combined with multi-index classifiers predict postoperative recurrence and survival of GBC (32). In this study, the combination of the MIC1 classifier and the “avNNet” model can predict recurrence with an AUC of 0.944, and a sensitivity and specificity of 0.857 and 1.000, respectively. The MIC2 classifier and “glmnet” model combination can predict survival with an AUC of 0.882, and a sensitivity and specificity of 0.765 and 1, respectively. These two models showed excellent performance in predicting the prognosis of GBC. Furthermore, Kaplan-Meier analysis of MIC1 and MIC2 indicators shows their performance in predicting DFS and OS of GBC. Eight of the ten MIC1 indicators can effectively predict the median survival of DFS, and there is no statistically significant difference in the prediction results of the eight indicators (χ^2 ^= 6.849, *P* = 0.653). In addition, seven of the nine MIC2 indicators can effectively predict the median survival of OS, and there is no statistically significant difference in the prediction results of the seven indicators (χ^2 ^= 9.14, *P* = 0.519).

The study of GBC is often limited by the difficulty of collecting clinical samples due to the low incidence. In addition, because of some unavoidable confounding factors, we did not contain sufficient information on participants in the sample, such as more long-term survival and outcome. In the future, further validation is needed to build up the fundamental basis for clinical application. We will expand alignment to validate the two models. Moreover, systematic prospective studies should be designed in sizeable multi-center sample cohorts, and the performance of these two prediction models should be studied more deeply and systematically.

## 5 Conclusion

Based on the clinical data of 122 patients, two multi-index classifiers, MIC1 and MIC2, were produced for predicting postoperative recurrence and survival of GBC by correlation analysis, relative risk analysis, ROC analysis, and AI algorithm Modeling. The MIC1 and MIC2 combined with avNNet and glmnet models to predict recurrence and survival were evaluated with high sensitivity and specificity. The prediction ability of indicators in the two classifiers to DFS and OS is assessed by Kaplan-Meier analysis. As a cheap, simple, reliable, and repeatable method, clinical multi-index analysis can be used to predict the prognosis of GBC in clinical practice. We aim to improve the postoperative prognosis management of GBC through these two classifier models and provide personalized treatment monitoring to improve the survival rate of GBC. Our findings may offer an attractive strategy for the prognostic management of GBC.

## Data availability statement

The original contributions presented in the study are included in the article/**Supplementary Material**. Further inquiries can be directed to the corresponding authors.

## Ethics statement

The studies involving human participants were reviewed and approved by the Ethics Committee of the Zhejiang Cancer Hospital. Written informed consent for participation was not required for this study in accordance with the national legislation and the institutional requirements.

## Author contributions

(I) Conception and design: YZ. (II) Administrative support: SC, YC, and SX. (III) Provision of study materials or patients and collection and assembly of data: YW. (IV) Data analysis and interpretation and manuscript writing: LL and QL. (V) Final approval of manuscript: All authors. All authors approved the final version of the manuscript. All authors contributed to the article.
