# Supplementary material for: Multi-clinical index classifier combined with AI algorithm model to predict the prognosis of gallbladder cancer
Source: Front Oncol. 2023 May 10;13:1171837. doi: 10.3389/fonc.2023.1171837 (PMC10206143; doi:10.3389/fonc.2023.1171837)
Supplement: Supplementary file 1 [file DataSheet_1.docx]

**Appendix：**

**Table S1. Clinical characteristics of enrolled patients with GBC.**

| Characteristics | N(%) | P(95%(CI)) |
| --- | --- | --- |
| WBC |  |  |
| ≥ 6.00 | 55 (0.4508) | 0.016 (−0.378 - −0.04) |
| < 6.00 | 67 (0.5492) |  |
| Neutrophil |  |  |
| ≥ 3.89 | 48 (0.3934) | 0.012 (−0.39 - −0.05) |
| < 3.89 | 74 (0.6066) |  |
| PCT |  |  |
| ≥ 0.24 | 53 (0.4344) | 0.042 (−0.348 - −0.007) |
| < 0.24 | 69 (0.5656) |  |
| Globulin |  |  |
| ≥ 27.95 | 51 (0.4180) | 0.319 (−0.263 - 0.086) |
| < 27.95 | 71 (0.5820) |  |
| MPV |  |  |
| ≥ 10.60 | 70 (0.5738) | 0.032 (−0.361 - −0.016) |
| < 10.60 | 52 (0.4262) |  |
| GGT |  |  |
| ≥ 120.00 | 30 (0.2459) | 0.009 (−0.432 - −0.065) |
| < 120.00 | 92 (0.7541) |  |
| TBIL |  |  |
| ≥ 30.97 | 16 (0.1311) | 0.03 (−0.49 - −0.028) |
| < 30.97 | 106 (0.8689) |  |
| PHOS |  |  |
| ≥ 1.13 | 67 (0.5492) | 0.103 (−0.317 - 0.029) |
| < 1.13 | 55 (0.4508) |  |
| ALP |  |  |
| ≥ 119.00 | 34 (0.2787) | 0.005 (−0.433 - −0.078) |
| < 119.00 | 88 (0.7213) |  |
| Fibrinogen |  |  |
| ≥ 3.38 | 50 (0.4063) | 0.000 (−0.56 - −0.252) |
| < 3.38 | 72 (0.5938) |  |
| TRFA |  |  |
| ≥ 0.08 | 42 (0.3516) | 0.000 (−0.522 - −0.206) |
| < 0.08 | 80 (0.6484) |  |
| CA199 |  |  |
| ≥ 772.00 | 20 (0.1641) | 0.000 (−0.546 - −0.217) |
| < 772.00 | 102 (0.8359) |  |
| CA125 |  |  |
| ≥ 31.50 | 24 (0.2031) | 0.000 (−0.537 - −0.213) |
| < 31.50 | 98 (0.7969) |  |
| Ferritin |  |  |
| ≥ 296.25 | 41 (0.3281) | 0.000 (−0.495 - −0.17) |
| < 296.25 | 81 (0.6719) |  |
| Cl |  |  |
| ≥ 103.16 | 76 (0.6171) | 0.018（−0.379 - −0.037） |
| < 103.16 | 46 (0.3828) |  |
| Na |  |  |
| ≥ 141.55 | 67 (0.5313) | 0.035（−0.354 - −0.013） |
| < 141.55 | 55 (0.4688) |  |
| ALT |  |  |
| ≥ 41.84 | 25 (0.2031) | 0.081（−0.023 - 0.387） |
| < 41.84 | 97 (0.7969) |  |
| GGT/ALB |  |  |
| ≥ 2.94 | 30 (0.2344) | 0.014（0.049 - 0.423） |
| < 2.94 | 92 (0.7656) |  |

WBC: white blood cell count; PCT: thrombocytocrit; MPV: mean platelet volume; GGT: gamma-glutamyl transpeptidase; TBIL: total bilirubin; ALP: alkaline phosphatase; TRFA: fibrinogen/albumin; CA125: carbohydrate antigen 125; CA199: carbohydrate antigen 199; Cl: chlorine; Na: Sodium; ALT: alanine transaminase; GGT/ALB: gamma-glutamyl transpeptidase/albumin.

**Table S2. Correlation between recurrence and clinical characteristics.**

| Factor | Mean | SD | P | 95% CI | |
| --- | --- | --- | --- | --- | --- |
|  |  |  |  | Low | High |
| Scope of surgery | 1.09 | 0.336 | 0.006 | 0.038 | 0.224 |
| Site | 1.65 | 0.479 | 0.010 | -0.343 | -0.049 |
| PVI, HAI | 1.91 | 0.293 | 0.007 | -0.161 | -0.026 |
| T | 1.47 | 0.502 | 0.000 | -0.579 | -0.299 |
| N | 1.40 | 0.493 | 0.000 | -0.567 | -0.256 |
| TNM | 1.21 | 0.412 | 0.000 | -0.708 | -0.412 |
| LNM | 1.59 | 2.150 | 0.000 | 0.754 | 1.816 |
| VI | 1.73 | 0.445 | 0.003 | -0.316 | -0.066 |
| NI | 1.71 | 0.458 | 0.003 | -0.331 | -0.067 |
| CN | 1.95 | 0.226 | 0.045 | -0.105 | -0.001 |
| MNC | 0.4243 | 0.18452 | 0.013 | 0.01647 | 0.13923 |
| Neutrophil | 4.1733 | 1.73901 | 0.019 | 0.11587 | 1.24967 |
| PCT | 0.2525 | 0.07946 | 0.008 | 0.00973 | 0.06364 |
| DBIL | 28.9320 | 66.46588 | 0.005 | 7.21714 | 38.26196 |
| AST | 39.5333 | 42.16901 | 0.011 | 3.59096 | 26.75873 |
| GGT | 162.7600 | 250.31282 | 0.003 | 35.17944 | 169.39716 |
| ALT | 52.8667 | 70.39432 | 0.011 | 6.31264 | 46.93013 |
| Cl | 102.6667 | 2.87267 | 0.018 | -2.36124 | -0.22996 |
| TBIL | 42.9893 | 82.06429 | 0.003 | 9.88318 | 48.17850 |
| ALP | 145.0400 | 119.22525 | 0.000 | 32.19849 | 89.20226 |
| TBA | 32.5293 | 64.09857 | 0.044 | 0.53194 | 37.31918 |
| FIB | 3.6552 | 1.12816 | 0.001 | 0.26576 | 1.07030 |
| TRFA | 0.0884 | 0.03516 | 0.003 | 0.00640 | 0.03076 |
| CA199 | 1148.3267 | 2733.85928 | 0.013 | 199.42637 | 1616.63451 |
| CA125 | 39.2760 | 55.59005 | 0.022 | 2.80160 | 34.70512 |
| FER | 369.0160 | 385.59135 | 0.001 | 71.44222 | 280.01997 |

PVI: portal vein invasion; HAI: hepatic artery invasion; T: T-stage; N: N-stage; M: M-stage; TNM: TNM-stage; LNM: lymph node metastasis; VI: vascular invasion; NI: nerve invasion; CN: cancer node; MNC: mononuclear cell; PCT: thrombocytocrit; DBIL: direct bilirubin; AST: glutamic oxalacetic transaminase; GGT: gamma-glutamyl transpeptidase; ALT: alanine transaminase; Cl: chlorine; TBIL: total bilirubin; ALP: alkaline phosphatase; TBA: total bile acid; FIB: fibrinogen; TRFA: fibrinogen/albumin; CA199: carbohydrate antigen 199; CA125: carbohydrate antigen 125; FER: ferritin.

**Table S3. Correlation between survival and clinical characteristics.**

| Factors | Mean | SD | P | 95% CI | |
| --- | --- | --- | --- | --- | --- |
|  |  |  |  | Low | High |
| Scope of surgery | 0.970 | 0.174 | 0.013 | -0.301 | -0.037 |
| T | 1.939 | 0.242 | 0.000 | 0.335 | 0.710 |
| N | 1.818 | 0.392 | 0.005 | 0.101 | 0.535 |
| TNM | 1.818 | 0.392 | 0.000 | 0.433 | 0.815 |
| LNM | 0.273 | 0.674 | 0.003 | -2.135 | -0.486 |
| RLN | 5.152 | 3.801 | 0.040 | -4.097 | -0.100 |
| NI | 1.939 | 0.242 | 0.004 | 0.092 | 0.454 |
| WBC | 5.239 | 1.279 | 0.016 | -1.785 | -0.192 |
| Neutrophil | 3.273 | 1.077 | 0.004 | -1.680 | -0.341 |
| MPV | 10.588 | 1.074 | 0.027 | -1.225 | -0.077 |
| GLB | 26.418 | 3.971 | 0.049 | -3.804 | -0.009 |
| GGT | 63.758 | 145.523 | 0.038 | -201.100 | -5.774 |
| GGT/ALB | 1.523 | 3.512 | 0.033 | -4.985 | -0.212 |
| Cl | 104.576 | 3.000 | 0.000 | 1.151 | 3.778 |
| Na | 142.152 | 2.575 | 0.015 | 0.304 | 2.777 |
| TBIL | 42.153 | 77.049 | 0.049 | -53.496 | -0.094 |
| PHOS | 1.062 | 0.215 | 0.008 | -0.220 | -0.034 |
| ALP | 82.030 | 32.406 | 0.003 | -110.869 | -23.459 |
| FIB | 2.888 | 0.954 | 0.001 | -1.378 | -0.358 |
| TRFA | 0.068 | 0.026 | 0.002 | -0.037 | -0.008 |
| FER | 142.385 | 110.053 | 0.001 | -298.673 | -82.824 |

T: T-stage; N: N-stage; TNM: TNM-stage; LNM: lymph node metastasis; RLN: regional lymph node; NI: nerve invasion; WBC: white blood cell count; MPV: mean platelet volume; GLB: globin; GGT: gamma-glutamyl transpeptidase; GGT/ALB: gamma-glutamyl transpeptidase/albumin; Cl: chlorine; Na: Sodium; TBIL: total bilirubin; ALP: alkaline phosphatase; FIB: fibrinogen; TRFA: fibrinogen/albumin; FER: ferritin.

**Table S4. Risk factors for recurrence in patients with GBC.**

| Risk factor | Relative risk (RR) | 95% CI | | P |
| --- | --- | --- | --- | --- |
|  |  | Low | High |  |
| TNM | 2.960 | 1.928 | 4.545 | 0.000 |
| T | 2.108 | 1.605 | 2.768 | 0.000 |
| N | 1.991 | 1.473 | 2.692 | 0.000 |
| LNM | 1.991 | 1.473 | 2.692 | 0.000 |
| FIB | 1.964 | 1.467 | 2.629 | 0.000 |
| TRFA | 1.796 | 1.370 | 2.354 | 0.000 |
| Scope of surgery | 1.791 | 1.528 | 2.100 | 0.021 |
| PVI, HAI | 1.779 | 1.520 | 2.083 | 0.041 |
| CA125 | 1.735 | 1.371 | 2.196 | 0.001 |
| CA199 | 1.729 | 1.376 | 2.171 | 0.001 |
| AST | 1.707 | 1.338 | 2.177 | 0.001 |
| FER | 1.698 | 1.302 | 2.215 | 0.000 |
| DBIL | 1.580 | 1.218 | 2.048 | 0.019 |
| TBA | 1.580 | 1.218 | 2.048 | 0.019 |
| VI | 1.576 | 1.221 | 2.033 | 0.006 |
| NI | 1.553 | 1.199 | 2.011 | 0.008 |
| ALP | 1.495 | 1.145 | 1.952 | 0.009 |
| GGT | 1.472 | 1.126 | 1.925 | 0.015 |
| TBIL | 1.468 | 1.100 | 1.958 | 0.049 |
| Site | 1.467 | 1.121 | 1.920 | 0.015 |
| Neutrophil | 1.440 | 1.087 | 1.908 | 0.014 |
| PCT | 1.349 | 1.010 | 1.802 | 0.043 |

TNM: TNM-stage; T: T-stage; N: N-stage; LNM: lymph node metastasis; FIB: fibrinogen; TRFA: fibrinogen/albumin; PVI: portal vein invasion; HAI: hepatic artery invasion; CA125: carbohydrate antigen 125; CA199: carbohydrate antigen 199; AST: glutamic oxalacetic transaminase; FER: ferritin; DBIL: direct bilirubin; TBA: total bile acid; VI: vascular invasion; NI: nerve invasion; ALP: alkaline phosphatase; GGT: gamma-glutamyl transpeptidase; TBIL: total bilirubin; PCT: thrombocytocrit.

**Table S5. Risk factors for survival in patients with GBC.**

| Risk factor | Relative risk (RR) | 95% CI | | P |
| --- | --- | --- | --- | --- |
|  |  | Low | High |  |
| T | 2.800 | 1.814 | 4.322 | 0.000 |
| N | 1.875 | 1.225 | 2.871 | 0.006 |
| TNM | 4.024 | 2.045 | 7.921 | 0.000 |
| LNM | 1.875 | 1.225 | 2.871 | 0.006 |
| RLN | 1.848 | 1.194 | 2.862 | 0.007 |
| NI | 1.964 | 1.359 | 2.840 | 0.005 |
| WBC | 1.724 | 1.098 | 2.707 | 0.017 |
| Neutrophil | 1.637 | 1.050 | 2.552 | 0.031 |
| MPV | 2.046 | 1.255 | 3.334 | 0.002 |
| GGT | 1.601 | 1.056 | 2.430 | 0.048 |
| PHOS | 1.719 | 1.053 | 2.805 | 0.022 |
| ALP | 1.675 | 1.109 | 2.529 | 0.027 |
| FIB | 2.301 | 1.442 | 3.671 | 0.000 |
| TRFA | 2.067 | 1.329 | 3.215 | 0.001 |
| CA125 | 1.964 | 1.359 | 2.840 | 0.005 |
| FER | 1.875 | 1.225 | 2.871 | 0.006 |

T: T-stage; N: N-stage; TNM: TNM-stage; LNM: lymph node metastasis; RLN: regional lymph node; NI: nerve invasion; WBC: white blood cell count; MPV: mean platelet volume; GGT: gamma-glutamyl transpeptidase; ALP: alkaline phosphatase; FIB: fibrinogen; TRFA: fibrinogen/albumin; CA125: carbohydrate antigen 125; FER: ferritin.

**Table S6. Estimated value of mean and median of DFS by MIC1.**

|  | Mean | | | | Median | | | | χ^2^ | P |
| --- | --- | --- | --- | --- | --- | --- | --- | --- | --- | --- |
| MIC1 indicators | Estimated value | SD | 95% CI | | Estimated value | SD | 95% CI | |  |  |
|  |  |  | Low | High |  |  | Low | High |  |  |
|  |  |  |  |  |  |  |  |  |  |  |
| CA199 | 20.294 | 7.078 | 6.422 | 34.166 | 8 | 2.395 | 3.305 | 12.695 | 14.971 | 0.000 |
| T | 16.275 | 2.864 | 10.661 | 21.889 | 11 | 2.135 | 6.815 | 15.185 | 31.628 | 0.000 |
| N | 22.554 | 3.626 | 15.447 | 29.661 | 12 | 1.223 | 9.603 | 14.397 | 21.470 | 0.000 |
| TNM | 21.081 | 3.078 | 15.049 | 27.113 | 12 | 1.173 | 9.702 | 14.298 | 38.707 | 0.000 |
| FIB | 27.112 | 4.553 | 18.188 | 36.036 | 12 | 1.880 | 8.314 | 15.686 | 20.007 | 0.000 |
| LNM | 22.554 | 3.626 | 15.447 | 29.661 | 12 | 1.223 | 9.603 | 14.397 | 21.471 | 0.000 |
| VI | 22.112 | 5.144 | 12.030 | 32.194 | 13 | 3.475 | 6.190 | 19.810 | 6.293 | 0.012 |
| TRFA | 26.08 | 3.686 | 18.856 | 33.304 | 14 | 3.611 | 6.922 | 21.078 | 13.610 | 0.000 |
| GGT | 26.307 | 4.88 | 16.742 | 35.872 | 13 | 3.913 | 5.330 | 20.67 | 2.881 | 0.090 |
| ALP | 30.129 | 4.679 | 20.959 | 39.3 | 17 | 10.927 | 0.000 | 38.417 | 1.378 | 0.240 |

CA199: carbohydrate antigen 199; T: T-stage; N: N-stage; TNM: TNM-stage; FIB: fibrinogen; LNM: lymph node metastasis; VI: vascular invasion; TRFA: fibrinogen/albumin; GGT: gamma-glutamyl transpeptidase; ALP: alkaline phosphatase.

**Table S7. Estimated value of mean and median of OS by MIC2.**

| MIC2 indicators | Mean | | | | Median | | | | χ^2^ | P |
| --- | --- | --- | --- | --- | --- | --- | --- | --- | --- | --- |
|  | Estimated value | SD | 95% CI | | Estimated value | SD | 95% CI | |  |  |
|  |  |  | Low | High |  |  | Low | High |  |  |
| T | 23.802 | 4.188 | 15.593 | 32.010 | 21.000 | 2.746 | 15.617 | 26.383 | 23.630 | 0.000 |
| TNM | 26.076 | 3.617 | 18.986 | 33.165 | 22.000 | 3.407 | 15.322 | 28.678 | 28.997 | 0.000 |
| LNM | 27.986 | 4.559 | 19.050 | 36.922 | 22.000 | 6.124 | 9.998 | 34.002 | 11.220 | 0.001 |
| NI | 28.964 | 4.979 | 19.205 | 38.724 | 22.000 | 2.806 | 16.500 | 27.500 | 6.069 | 0.014 |
| FIB | 31.139 | 5.024 | 21.293 | 40.986 | 22.000 | 2.619 | 16.868 | 27.132 | 13.202 | 0.000 |
| MPV | 34.183 | 4.647 | 25.075 | 43.292 | 25.000 | 4.108 | 16.948 | 33.052 | 9.573 | 0.002 |
| TRFA | 32.132 | 4.336 | 23.634 | 40.630 | 25.000 | 4.371 | 16.432 | 33.568 | 6.691 | 0.010 |
| Neutrophil | 38.660 | 5.540 | 27.802 | 49.519 | 25.000 | 12.116 | 1.253 | 48.747 | 3.176 | 0.075 |
| GGT | 40.139 | 4.966 | 30.405 | 49.872 | 39.000 | 7.714 | 23.880 | 54.120 | 0.018 | 0.893 |

T: T-stage; TNM: TNM-stage; LNM: lymph node metastasis; NI: nerve invasion; FIB: fibrinogen; MPV: mean platelet volume; TRFA: fibrinogen/albumin; GGT: gamma-glutamyl transpeptidase.
